# Efficacy of Platelet-Rich Plasma in Males With Androgenetic Alopecia

**DOI:** 10.7759/cureus.36531

**Published:** 2023-03-22

**Authors:** Navakumar Manickam, Prashant Mathapati, Keerthana Somasundaram, Kannan Gopalan, Seethalakshmi Ganga Vellaisamy

**Affiliations:** 1 Dermatology, Vinayaka Mission's Kirupananda Variyar Medical College and Hospital, Salem, IND; 2 Dermatology, Vinayaka Mission’s Kirupananda Variyar Medical College and Hospital, Salem, IND

**Keywords:** males, prp, platelet rich plasma, improvement, androgenetic alopecia

## Abstract

Background: Androgenetic alopecia (AGA) is a common cause of hair loss in men with limited treatment options. Platelet-rich plasma (PRP) therapy is one of the newer treatment modalities in the management of AGA with promising results.

Aim: The aim is to assess the efficacy of PRP in males with AGA and to study any adverse events associated with the procedure.

Methods: A total of 30 patients participated in the study, and they were administered PRP injections every three weeks for four sessions. An evaluator’s visual assessment of improvement based on digital photographs was done at three, six, and nine weeks and then at the end of the fourth month. The patient’s perception of improvement was evaluated on a 7-point scale at the baseline and at the end of the fourth month.

Results: Of the 30 participants, 27 completed all four sessions. Mild improvement was seen in 13 (48.1%) patients, moderate improvement was seen in five (18.5%) patients, and excellent improvement was seen in one (3.7%) patient. Six (22.2%) patients had involvement in the vertex region of the scalp; of them, three (50%) had moderate improvement, one (16.7%) had excellent improvement, and two (33.3%) had mild improvement. Twenty-one (77.8%) patients had involvement in the fronto-temporal region of the scalp, of which nine (42.9%) had mild improvement, and five (23.8%) had moderate improvement.

Conclusion: PRP treatment alone appeared to be a simple, cost-effective treatment for AGA with good results.

## Introduction

Androgenetic alopecia (AGA) is characterized by progressive, patterned hair loss that occurs as a result of excessive sensitivity to androgens in genetically predisposed individuals [1]. AGA occurs due to the androgen-mediated conversion of terminal hairs to vellus hairs. It usually begins at 20 years of age and nearly 50% of males are affected by the age of 50 years [2]. There is an increase in the frequency and severity of AGA with age [3]. It is characterized by the gradual thinning of scalp hairs in a defined pattern, causing a significant lowering of self-esteem and psychological well-being of the individual [2].

Currently, the most promising treatments available for the management of AGA are drugs such as minoxidil and finasteride. Drugs are not always effective, take a long time to show their effective results, and finasteride is notable for unacceptable side effects like sexual dysfunction. Hence, there is a need for adjuvant and newer treatment modalities that give faster and better responses [1]. Hair transplantation is also a very tedious procedure. Platelet-rich plasma (PRP) is widely used in various medical and surgical specialties due to its ability to improve tissue repair and healing [4].

PRP is an autologous preparation of platelets in concentrated plasma, which usually contains platelets in a higher concentration than the normal range. It is an interesting, safe, easy, and inexpensive modality to treat AGA, with no risk of allergic reactions. Activated platelet alpha granules release various growth factors, which promote new hair growth through various mechanisms such as the proliferation of dermal papillae cells, thickening of pre-existing hair shafts, and faster transformation of telogen to anagen phase [5]. Studies related to PRP in AGA are limited in number, which prompted us to take up this research to assess the efficacy of PRP in AGA and to study any adverse effects associated with the procedure.

## Materials and methods

**Figure 1:**
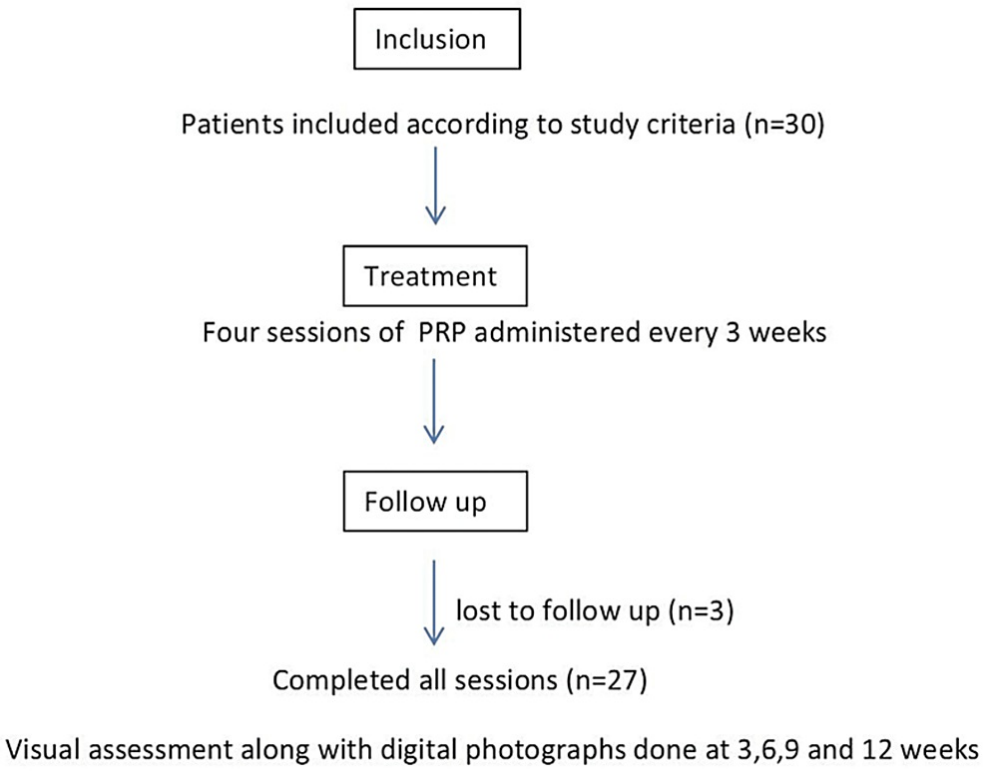
Flow chart of the study

This was a prospective unicentric observational study conducted over a period of one year from January 2019 to January 2020 among 30 male patients with AGA. The sample size was calculated using the formula for population proportion with the power of study of 80% and precision alpha of 0.05 with a 95% confidence interval. The study was approved by the institutional ethical committee (VMKVMC/IEC/18/42) and informed written consent was obtained from all participants. The diagnosis of AGA was made on a clinical basis based on the detailed history and examination findings. The staging of AGA was done according to the modified Norwood-Hamilton scale [6]. Major inclusion criteria were all male patients from 18 to 50 years of age with AGA grading II to V on the Modified Norwood Hamilton scale and those who did not undergo any treatment in the past six months with the exclusion criteria being patients with coagulopathies, current treatment with anticoagulants or anti-platelets medication, other types of alopecia like alopecia areata, cicatricial alopecias, etc. keloidal tendencies, coexisting dermatoses like psoriasis or lichen planus due to the risk of Koebner phenomenon and those on intake of any drugs known to cause alopecias. Prior to the procedure, all patients had a baseline blood workup that included a complete blood count, coagulation and blood sugar profile, HIV, Hepatitis B, and Hepatitis C screening. All patients were given four sessions of PRP injections every three weeks. A visual assessment along with a digital photograph was done at three, six, and nine weeks and then at the completion of the fourth month. The flow chart of the study is shown in Figure 1.

Parameters for assessments

1. Patient’s self-assessment of improvement on a 7-point scale (Table 1) [1].

**Table 1:** Patient’s self-assessment of improvement on a 7-point scale

Score	Improvement
-3	Severe hair fall
-2	Moderate hair fall
-1	Mild hair fall
0	No change
+1	Mild improvement
+2	Moderate improvement
+3	Excellent improvement

2. Visual assessment of improvement based on digital photographs by a blinded independent senior dermatologist on a 7-point scale (Table 2) [1].

**Table 2:** Evaluator’s assessment of improvement on a 7-point scale

Score	Improvement
-3	Severe worsening
-2	Moderate worsening
-1	Mild worsening
0	No change
+1	Mild improvement
+2	Moderate improvement
+3	Excellent improvement

3. A hair pull test was done at baseline and at each visit. It was performed by pulling around 20-60 hairs firmly grasped between the thumb, index, and middle fingers from the base of the hairs tugged away from the scalp [7]. The test was considered positive if there were more than 10% of hairs pulled away from the scalp, which indicates active hair fall.

PRP procedure

Under aseptic precautions, around 16ml of peripheral blood was collected from the median cubital vein and transferred into a sodium citrate vacutainer. The tubes were rotated in a centrifuge machine (REMI) at 2,000 revolutions per minute for 6 minutes. This first centrifugation is called “soft spin,” which separates the blood into three layers: the lowermost RBC layer; the topmost acellular plasma layer called platelet poor plasma; and an intermediate PRP layer called the buffy coat. The Buffy coat along with the plasma was collected with the help of a pipette in another test tube. This tube was again subjected to a second centrifugation at 4000 revolutions per minute for 10 minutes, called “hard spin.” This allows the platelets to settle at the bottom of the tube. The upper layer containing platelet poor plasma was discarded, and the lower layer of the PRP was collected in another clean tube. The platelet concentrate was loaded into an insulin syringe containing calcium chloride (nine parts of PRP and one part of calcium chloride) as an activator and then injected evenly into the affected areas of the scalp. Multiple PRP injections of 0.1 mL were given at each site in a linear pattern 1 cm apart using the nappage technique.

The data were entered into SPSS software for further analysis. Continuous variables were expressed as mean ± SD and categorical variables were expressed as numbers and percentages. Fisher’s exact test was used to analyze the qualitative data. P-value of less than 0.05 was considered to be statistically significant.

## Results

**Figure 2:**
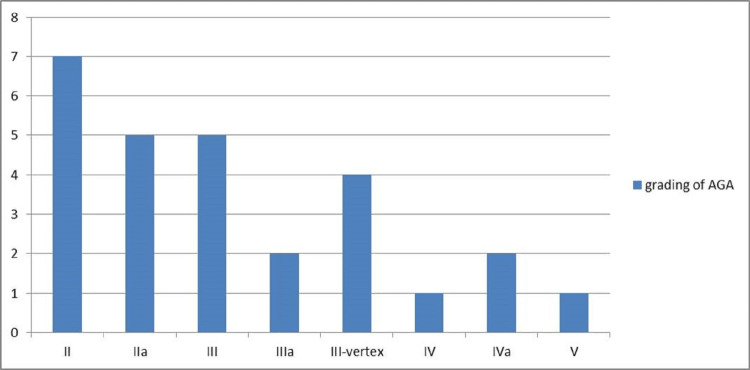
Distribution of the study population based on Modified Norwood Hamilton classification

Of the 30 participants, 27 (90%) completed all four sessions and three were lost to follow-up due to personal reasons. The mean age of the study population was 25.4±3.5 years. The duration of hair loss ranged from six months to seven years. The mean duration of hair loss in our study was 35.6 months. A significant family history was seen in 16 (59.3%) patients with AGA. Of the 27 patients, 23 (85.2%) had grade II-III and four (14.8%) had grade IV-V AGA. Figure 2 shows the distribution of the study population based on the modified Norwood-Hamilton classification.

**Figure 3:**
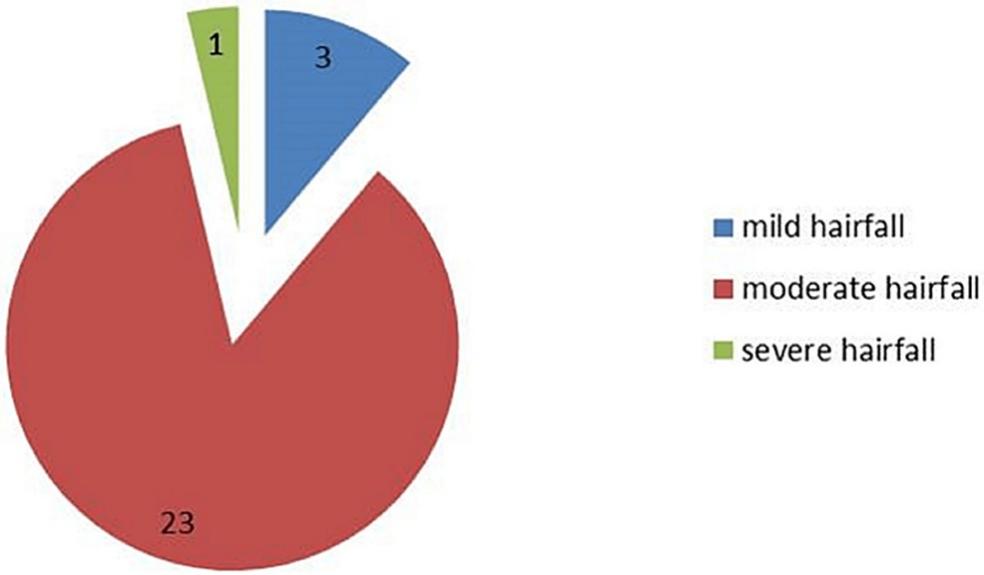
Patient’s assessment at baseline

The patient’s perception of improvement in the degree of hairfall and hair growth was evaluated on a 7-point scale at baseline and at the end of the fourth month. Figure 3 shows the patient’s assessment of hair fall at baseline.

Of the 27 patients, 22 (81.4%) had mild to moderate improvement, 1 (3.7%) had excellent improvement, and 4 (14.8%) had no change at the end of the fourth month (Table 3).

**Table 3:** Patient’s self-assessment of improvement on a 7-point scale at fourth month

	At 4^th^ month
0(no change)	4(14.8%)
+1(mild improvement)	11(40.7%)
+2(moderate improvement)	11(40.7%)
+3(excellent improvement)	1(3.7%)
Total	27(100%)

The evaluator assessed the digital photographs taken at baseline, at each visit (three, six, and nine weeks) and at the end of the fourth month on a standard 7-point scale, the results of which are shown in Table 4.

**Table 4:** Evaluator’s visual assessment of improvement on a 7-point scale

	3^rd^ week	6^th^ week	9^th^ week	4^th^ month
No change	27(100%)	22(81.5%)	10(37%)	7(25.9%)
Mild improvement	0	5(18.5%)	15(55.6%)	11(40.7%)
Moderate improvement	0	0	1(3.7%)	8(29.6%)
Excellent improvement	0	0	1(3.7%)	1(3.7%)

The evaluator’s visual assessment showed 19 (70.37%) patients with mild to moderate improvement and one (3.7%) patient with excellent improvement at the end of the fourth month. A hair pull test done to assess disease severity showed 15 (55.6%) patients with a positive test at baseline, which reduced to only one (3.7%) patient at the end of treatment, indicating good control of hair fall with PRP.

**Figure 4:**
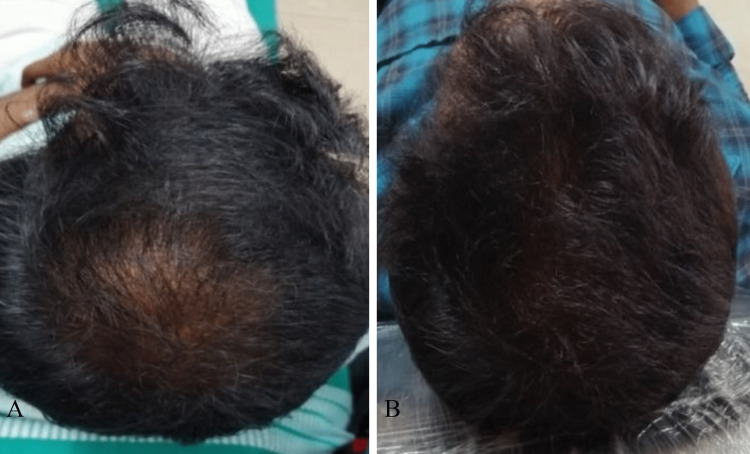
A 30-year-old male with grade III vertex AGA at baseline (A). Excellent improvement seen after four sessions of PRP (B).

The vertex region of the scalp was affected in six (22.2%) patients, of which three (50%) had moderate improvement, one (16.7%) had excellent improvement (Figures 4A, 4B), and two (33.3%) had mild improvement (Table 5).

 

**Table 5:** Evaluator’s assessment of improvement in patients involving vertex region

AGA grade	No change	Mild improvement	Moderate improvement	Excellent improvement	Total
III-vertex	0	0	3(75%)	1(25%)	4(100%)
IV	0	1(100%)	0	0	1(100%)
V	0	1(100%)	0	0	1(100%)

**Figure 5:**
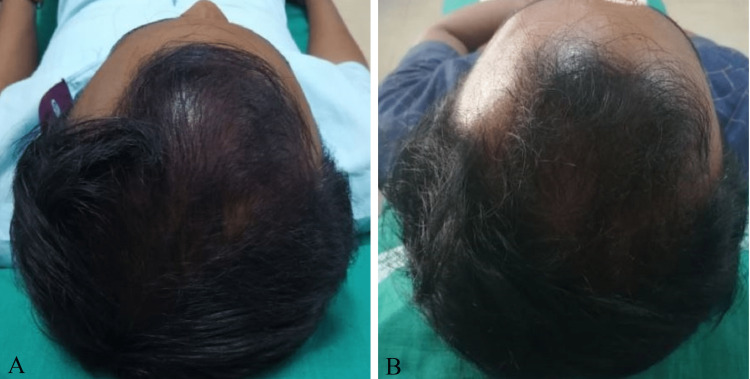
A 26-year-old male with grade III AGA at baseline (A). Moderate improvement seen after four sessions of PRP (B).

Twenty-one (77.8%) patients with involvement of the fronto-temporal region of the scalp showed mild improvement in nine (42.9%) patients, moderate improvement in five (23.8%) patients (Figures 5A, 5B), while seven (33.3%) patients had no improvement (Table 6).

 

**Table 6:** Evaluator’s assessment of improvement in patients involving fronto-temporal region of scalp

AGA grade	No change	Mild improvement	Moderate improvement	Excellent improvement
II (n=7)	1(14.3%)	4(57.1%)	2(28.6%)	0
IIa (n=5)	3(60%)	1(20%)	1(20%)	0
III (n=5)	2(40%)	3(60%)	0	0
IIIa (n=2)	1(50%)	0	1(50%)	0
IVa (n=2)	0	1(50%)	1(50%)	0
Total (n=21)	7(33.3%)	9(42.9%)	5(23.8%)	0

A comparative analysis of treatment responses between the vertex region and the fronto-temporal region of the scalp was done, which revealed that improvement was more frequent among patients with AGA involving the vertex region of the scalp. However, this was not statistically significant (Table 7).

**Table 7:** Evaluator’s assessment of improvement in vertex region vs fronto-temporal region of scalp

	Vertex	Fronto-temporal	P value
No change	0	7(33.3%)	0.15
Mild improvement	2(33.3%)	9(42.9%)	1.00
Moderate improvement	3(50%)	5(23.8%)	0.32
Excellent improvement	1(16.7%)	0	0.22
Total	6(100%)	21(100%)	

Side effects were minimal and observed only in five patients. Pain was the main complaint in three (11.1%) patients post procedure, which subsided with a single dose of paracetamol 500mg. Pruritus was reported in two (7.4%) patients, and it subsided spontaneously within a day.

## Discussion

AGA is the most common type of diffuse non-scarring alopecia seen in males. The FDA approved treatment options are topical minoxidil or oral finasteride, used alone or in combination. However, response to both these treatments has been unsatisfactory in AGA. Since AGA is characterized by miniaturization of terminal to vellus hairs and a shortened anagen phase, current treatment strategies target cellular proliferation and differentiation occurring during the hair cycle [8].

PRP has emerged as a new treatment option for AGA, and numerous new studies on the potential effect of PRP on hair have been published. One of the major contributing factors stimulating hair growth has been suggested to be the antiapoptotic effects of activated PRP [8,9]. Prolonged survival of dermal papilla cells during the hair cycle occurs due to PRP-induced activation of various antiapoptotic regulators, such as the Bcl-2 protein and Akt signalling [8,10]. PRP mediated upregulation of FGF-7/β-catenin signalling pathways promotes hair growth by inducing differentiation of follicular stem cells as well as prolonging the anagen phase of the hair cycle [8,11]. PRP also increases the formation of perifollicular vascular plexus by increasing the levels of angiogenic factors such as vascular endothelial growth factor (VEGF) and platelet derived growth factor (PDGF) [12].

In the present study, 13 (48.1%) patients had mild improvement, five (18.5%) patients had moderate improvement and one (3.7%) patient had excellent improvement with PRP treatment. All six (100%) patients with vertex involvement showed a response to PRP, as three (50%) patients had moderate improvement, two (33.3%) patients had mild improvement, and one (16.7%) patient had excellent improvement. In contrast, among 21 patients with involvement of the fronto-temporal region, five (23.8%) patients had moderate improvement, and nine (42.9%) patients had mild improvement, while seven (33.3%) patients had no response to treatment.

Among four patients with grade III-vertex involvement, three (75%) had moderate improvement and one (25%) had excellent improvement. Thus, we observed a better response in Grade III-vertex AGA compared to more advanced grades of alopecia. A comparison between the vertex and fronto-temporal regions of the scalp revealed a favorable treatment response among patients with AGA involving the vertex region, even though it was not statistically significant. This finding was comparable with previous studies by Gkini et al. [12] and Rodrigues et al. [13].

In our study, at baseline, 15 (55.6%) patients had positive hair pull test which reduced significantly at the fourth month with only one (3.7%) patient having a positive hair pull test and the remaining 26 (96.3%) patients turning negative to hair pull test. Gkini et al. [12] reported similar findings, with all patients showing negative hair pull test at three months after treatment.

Gkini et al. [12] reported a non-randomized trial to assess the efficacy of PRP injection in 20 patients affected by androgenic alopecia. PRP was prepared by single spin centrifugation at 1500g for 5 minutes. Patients were treated with three sessions of PRP at an interval of three weeks, and a booster session was also done at six months. Hair density significantly increased throughout the study period, with a peak at three months. They proposed that patients with Grade II-III AGA had a favorable treatment response compared to patients with more severe alopecia.

Schiavone et al. [14] studied 64 patients with AGA and reported the results obtained after two injections of leukocyte PRP (L-PRP) with the addition of concentrated plasmatic proteins, at baseline and after three months (single spin at baseline and double-spin centrifugation at three months). Two independent evaluators assessed the response by using the Jaeschke scale and clinical photographs. An improvement in global physician assessment score was seen in all patients by one evaluator and in 62 of 64 patients by the other evaluator.

Cervelli et al. [15] conducted a randomized, blinded study with a placebo half-head group in 10 patients with male AGA who were not currently on any other medication for the past 12 months and assessed the response by TrichoScan. PRP was obtained by centrifugation at 1,100 G for 10 minutes, and then activated with Ca2+ prior to administration. After three months, mean hair counts increased significantly by an average of 27.7 hairs/cm^2^ in the treated areas, compared to 3 hairs/cm^2^ in the control areas.

Singhal et al. [2] in a study of 10 patients with AGA administered PRP prepared by using double spin centrifugation. First spin was done at 1,500g for 6 minutes, followed by the second spin at 2,500g for 15 minutes. The study also had a control group, which was given medical treatment. PRP sessions were administered every 2-3 weeks for three months. Hair pull test done to assess response showed significant results, and an improvement in hair count and hair thickness was also noted.

Gentile et al. [8] conducted a randomized, evaluator-blinded, half-head-based, placebo-controlled trial including 20 male patients with AGA grade II to IV of the Norwood-Hamilton classification. PRP was prepared by centrifugation at 1,100 G for 10 minutes, and then activated with Ca2+. After three sessions of PRP at 30-day intervals, improvement in mean hair count and total hair density was seen in all patients compared with those who received placebo treatment.

Alves and Grimalt [16] performed a randomized, placebo-controlled, double-blind, half-head study in 25 male patients with AGA stages II-V of the Hamilton-Norwood scale. Patients were treated with three sessions of PRP, one month apart, and were evaluated using phototrichogram and global photography. At six months, a significant increase in mean anagen and telogen hairs, hair density, and terminal hair density was seen in PRP-treated areas compared with baseline. They proposed an application of three initial PRP sessions one month apart, followed by performing another three sessions of PRP after a six-month gap, with maintenance every six months or three sessions of PRP every year.

Rodrigues et al. [13] conducted a randomized, double-blind, placebo-controlled trial on 26 male patients with type III-vertex AGA for more than two years. PRP preparations were made using the single spin method with centrifugation at 1,258 G for 15 minutes. Patients treated with PRP were found to have a significant increase in hair density within three months after the last treatment, while the control group did not show a significant change in hair density.

Singh et al. [17] conducted a randomized, double-blind, placebo-controlled trial on 80 male patients with AGA type II-V according to the Norwood-Hamilton scale. PRP was prepared using the double spin method, with the first centrifugation at 2,200 rpm for 12 minutes, followed by the second centrifugation at 3,000 rpm for 6 minutes. The PRP obtained was then activated with 10% calcium gluconate. Dermoscopic images of an area of 1 cm^2^ were used for assessment by two independent evaluators. The PRP treatment group showed a significant improvement in hair density after receiving treatment for three months.

Zhou et al. [18] conducted a randomized, placebo-controlled trial on 10 male patients with type IIa-V AGA according to the Norwood-Hamilton classification. PRP was made using the double spin method with the first centrifugation at 200 G for 15 minutes followed by the second centrifugation at 1,200 G for 15 minutes. PRP was then activated with 10% calcium chloride. The PRP injected side showed a significant increase in mean hair density at three- and fourth-month follow-ups compared to placebo.

All of the abovementioned studies showed an improvement in hair growth and density after the PRP treatment, but the dose and administration protocol of PRP were not standardized. The evaluation methods used in each study were either too objective or too complicated to assess the improvement in daily clinical practice. The efficacy of PRP was assessed by multiple outcomes such as comparing before and after global photography, dermoscopic images, mean hair count, hair density, hair pull test, anagen-to-telogen ratio, and patient satisfaction surveys. In contrast, we evaluated the treatment response by subjective methods such as patient’s and evaluator’s self-assessment of improvement on a 7-point scale which is much simpler and convenient to use on a regular basis. Other factors such as the volume of whole blood withdrawn, centrifugation settings, and mean platelet enrichment also differed between the studies. This made our study difficult to compare with the previous studies.

The most common side effect seen in our study was temporary pain at the injection site, which was relieved with a single dose of paracetamol 500mg. A previous study by Dicle et al. [19] only reported mild side effects such as temporary pain and edema at the injection site, and they concluded that PRP therapy was relatively safe to use in AGA.

The major limitation of our study was the smaller sample size. A shorter follow up period of four months was another limiting factor, as some patients would have shown better improvement later, which might have been missed, and also, a longer follow-up would have been helpful in assessing the long-term efficacy of PRP. Evaluation of response in our study was subjective, unlike the objective methods used in previous studies. We did not measure the platelet counts obtained after centrifugation, as it would have assessed the effectiveness of our centrifugation method in yielding a good PRP concentration.

## Conclusions

PRP alone is a simple, cost-effective treatment modality for androgenic alopecia. Grade III vertex showed the best response among all types of AGA. Considering patients' high satisfaction rates with minimal adverse effects, PRP appears to be an effective alternative for patients who failed to show satisfactory responses to minoxidil or finasteride. PRP can also be combined with medications to further augment the treatment response. There is a need for large-scale randomized controlled trials with longer follow-up periods to evaluate the long-term sustainability of response achieved with PRP therapy.
